# Vulcanized Rubber

**Published:** 1860-01

**Authors:** 


### VULCANIZED RUBBER.

This material is being extensively introduced as a base for artificial dentures. In this city most of our dentists have taken hold of it and for the most part thus far seem to be well pleased with it, and all who have become adept in working it, are very much pleased with it in many respects. Of its valuable points we have spoken heretofore, and need not now repeat them. We now only wish to refer to a few things which progress seems to demand. The proper method of putting up the work is better understood than it was for sometime after its introduction ; there is more care and delicate manipulation employed than at first. Operators are becoming more familiar with the nature of the material, and the mode of using it.

From the limited experience which we have had with it, and from observation upon the mode in which the work is ordinarily done, we are convinced that more care is required to secure, or rather insure successful results than is usually given to it.

All new processes have to run the gauntlet of hasty, careless and unenlightened manipulation. This is an ordeal for which, those who introduce them are responsible. It is a strong inducement, with a majority of operators, to adopt a new process if it is easily and rapidly accomplished ; and usually the first attempts are most rapid, owing to an ardent desire to see a *result*.

The vulcanite base is just going through this ordeal, at least in the west, and it will be sometime before it will have recovered from that influence.

Real improvement is all the time being made by those most skillful and careful in its construction. The appliances and instruments are constantly being improved in efficiency and convenience, as well as being made cheaper. This is especially the case with the vulcanizing apparatus ; two years ago the apparatus cost about one hundred dollars. Then it was a large inconvenient affair requiring a good sized room for its accommodation. Now it is reduced to one-fifth that price, and nearly as much in size ; is a small, neat affair, that will be ornamental in an operating room ; and requires very little attention for its perfect control. With the improved apparatus, and the additional care that is being exercised in constructing it, we hope ere long to see the defects that have hitherto characterized it obliterated. The points in this work where defects have been most apparent are the arrangement and antagonism of the teeth, and the finish. The first of these requires great care to obtain proper results. As much care is required in the first arrangement as in any other method, and more care is required to keep them in proper position during the process than in any other style of work. Quite as much care and labor is required to give a fine finish to rubber work as any other, and patient labor is well rewarded too. This work will soon be in very general use. T.

